# How Partisan Policies Can Shape Health Behaviors: Executive Order Proof-of-Vaccine Mandate Bans Increased COVID-19 Vaccinations

**DOI:** 10.3390/vaccines14060486

**Published:** 2026-05-29

**Authors:** Deena N. Brosi, Gregory Tung, Beth M. McManus, Srinivas Parinandi, Glen P. Mays

**Affiliations:** 1 Department of Surgery, University of Colorado Anschutz Medical Campus, Aurora, CO 80045, USA; 2 Department of Health Systems, Management, and Policy, Colorado School of Public Health, Aurora, CO 80045, USA; 3 Political Science Department, University of Colorado at Boulder, Boulder, CO 80309, USA

**Keywords:** COVID-19 vaccination behavior, proof-of-vaccine mandate bans, psychological reactance theory, expressive function of law

## Abstract

Background/Objectives: COVID-19 vaccine resistance was detrimental to herd immunity and worsened COVID-19 morbidity and mortality during outbreaks. Despite more evidence showing reactionary behavior among residents exposed to vaccine mandates, little research has been conducted on the effects of state proof-of-vaccine (POV) mandate bans in the United States (US). We sought to investigate the causal effects of POV mandate bans, overall and stratified by policy passage via executive order or state legislature, on first-dose COVID-19 vaccinations. Methods: In the contiguous US, 21 states enacted POV mandate bans from 8 February 2021–25 October 2021. Using a geographic regression discontinuity design, we selected treatment and control counties within 150 miles of the POV mandate ban state border. The resulting sample was 4612 county-observations and 2466 unique counties. We conducted two-way fixed-effects estimation to compare changes in weekly, first-dose COVID-19 vaccinations among individuals <65 years old before and after POV mandate ban enactment between treatment and control counties. Results: Among executive order POV mandate ban counties, we saw an additional increase in weekly, first-dose COVID-19 vaccinations following POV mandate ban enactment when compared to controls. There was an additional 38.2% increase in Weeks 1–2, 40.6% in Weeks 3–4, 41.3% in Weeks 5–6, and 43.9% in Weeks 7–8. Conclusions: While seemingly counterintuitive, these findings follow Psychological Reactance Theory. Once the perceived threat to freedom was removed, reactance to COVID-19 vaccinations declined and constituents received the COVID-19 vaccine of their own volition. Future public health efforts should consider potential reactance to mandatory policies and tailor efforts to community values.

## 1. Introduction

After a comparatively poor public health response to the COVID-19 pandemic in 2020, the United States (US) rolled out the first ever mRNA vaccine for COVID-19 in early 2021 [1]. However, some Americans met the COVID-19 vaccine rollout with intense skepticism. In March 2021, a US Department of Health and Human Services (HHS) survey found that 19% of respondents reported they were “definitely not” or “probably not” going to receive the COVID-19 vaccine [2]. Skepticism was highest among those with a high school-level education, conservative political ideologies, and persons under 65 years old [2,3]. In response, the White House and federal agencies began discussing the potential for vaccine mandates. Several were eventually implemented in the Summer and Fall of 2021, including a Biden executive order requiring all healthcare workers to receive the COVID-19 vaccine and an Occupational Health and Safety Administration directive on employee vaccine mandates for large employers [4,5].

During those early White House discussions, news sources increased media content around the merits and consequences of vaccine mandates. This further politicized the topic, as demonstrated by the title of an opinion article from now Vice President JD Vance; “Vaccine passports are a terrible idea that deprive American families of basic choices” [1,6]. The result was a flurry of policy activity directed at vaccine mandates and passports at the state and local level. States like New York (NY) and California (CA) passed vaccine mandates for healthcare workers and state government employees. However, conservative states reacted in opposition, with some adopting proof-of-vaccine (POV) mandate bans. These POV mandate bans prohibited mostly government organizations, and in some cases private businesses, from requiring COVID-19 vaccination status for employees or patrons. By September 2021, twenty-two states had adopted some variation of the POV mandate ban [7].

Many researchers have tried to study the effects of vaccine mandates in the US, but no such research has been conducted on the effects of POV mandate bans on vaccination behavior [5]. Vaccine mandates, especially in other countries where the mandates extended to the general population, were effective at increasing COVID-19 vaccinations [8,9]. In the US, however, most vaccine mandates had little general-population effect, primarily because mandates only extended to certain populations (i.e., healthcare workers, state governments) and were implemented in states with already high COVID-19 vaccination rates [5,10]. Meanwhile, POV mandate bans were enacted in states with the highest unvaccinated populations and were seemingly intended to be more politically symbolic than a disincentive for vaccinating. POV mandate ban language emphasized individual liberties and the importance of state protections for those constitutional rights, all under the context of potential national COVID-19 vaccine mandates.

Given that COVID-19 vaccination rates among states with POV mandate bans were among the lowest in the country, it is important to understand the potential causal effects of this policy on COVID-19 vaccination behavior.

## 2. Materials and Methods

### 2.1. Conceptual Frameworks

**Figure 1:**
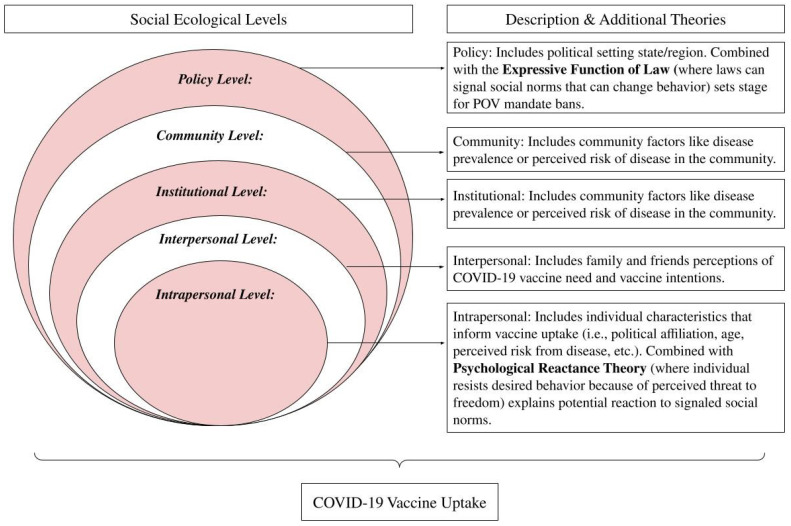
Full Social Ecological Model diagram with Expressive Function of Law and Psychological Reactance Theory falling under the policy and intrapersonal levels, respectively.

We used the Social Ecological Model (SEM) to guide our analysis of POV mandate ban effects on COVID-19 vaccinations (Figure 1). The SEM details how perceptions go beyond individual characteristics and that behavior change is a function of various social structures. For example, the SEM could consider how an individual’s political preference interacts with the community’s public health values when measuring intent to receive the COVID-19 vaccine [11]. It is under this framework, and with several complementary theories, that we set the stage for examining POV mandate ban enactment on COVID-19 vaccination behaviors.

By Spring 2021, studies showed that politically conservative areas were questioning the severity of the COVID-19 pandemic, and these perceptions were correlated with low COVID-19 vaccination rates [3]. Further, it was well documented during the COVID-19 pandemic that mandates, both mask and vaccine, produced behaviors described in *Psychological Reactance Theory* (PRT) [12,13,14]. PRT posits that individuals will resist desired behaviors if they believe it poses a threat to their freedom to choose [12,15]. Studies in the US and Germany documented higher reactance to mandatory COVID-19 vaccines, particularly among groups that were opposed to mandatory vaccinations prior to enactment [16,17]. In addition, studies among college students found that reactance to COVID-19 vaccination was higher among students posed with a hypothetical vaccine mandate at another university vs. at their university (coined indirect threat) [17]. We believe that conservative populations were experiencing this indirect reactance by observing vaccine mandate enactment in liberal states and hearing discussions of national vaccine mandates prior to POV mandate ban enactment. PRT studies also describe varying degrees of reactance behavior, which was tied to individual characteristics, such as beliefs around freedom of choice and political party affiliation [17,18,19]. Areas in the US that were more conservative and politically polarized would likely have stronger reactance to news around forced COVID-19 vaccinations and vaccine mandates [20,21,22].

This context is important when incorporating our next theory, the Expressive Function of Law (EFL). The EFL demonstrates the potential for policy, aside from the direct regulatory power and/or in the absence of enforcement, to change attitudes around a topic and motivate behavior change by signaling social norms [23]. For example, many localities did not enforce their new laws around mandatory recycling, but these laws signaled social expectations for residents, which increased recycling behavior. The EFL also theorizes that norms signaled through laws are most effective at changing behavior when it complements existing community values [23]. Many characteristics of POV mandate bans, including that they were largely unenforced and signaled the strongly held value of individual liberties to conservative constituents, fit within the EFL framework.

Under the SEM, EFL, and PRT, we present two hypotheses for POV mandate ban enactment, signaled norms, and subsequent COVID-19 vaccination behavior change. (1) POV mandate bans signaled that the COVID-19 pandemic was not as severe as federal public health institutions claimed. We hypothesize that this signaled norm would lead to relative decreases in COVID-19 vaccinations (compared to controls), particularly among populations who were not eager to vaccinate but would be persuaded if perceived risk of infection and disease repercussions were high [24]. (2) POV mandate bans signaled to a highly reactant population that an individual’s choice to vaccinate was now protected under the POV mandate ban. Under this scenario, we hypothesize a relative increase in COVID-19 vaccinations among more conservative and vaccine-resistant populations as the POV mandate bans relieve perceived threats to freedom of choice in vaccinating.

The first hypothesis is conceptually more straightforward. Many news outlets and public health officials criticized the enactment of POV mandate bans as being dangerous for COVID-19 vaccine perceptions and adoption [25,26,27]. While the second hypothesis may seem counterintuitive, it follows an extensive body of research around mitigating reactance behavior [15,19]. One particular study showed that participants with high reactance to messages promoting recycling reported more resistance to arguments and lower intention to recycle. However, once they were exposed to “restoration messaging” (i.e., “The choice is yours”), respondents had lower perceived threat to freedoms, more positive perceptions of recycling, and higher intent to recycle [28]. When applied to our analysis, POV mandate bans restored the perceived freedom of choice that reactant constituents believed was under threat and increased COVID-19 vaccinations as messaging around the importance of vaccinating remained.

### 2.2. Data and Measures

We obtained state-level POV mandate ban legislation and dates of enactment from the National Academy for State Health Policy (NASHP) website. By the end of 2021, 21 states in the contiguous US passed a POV mandate ban [7]. A list of state POV mandate bans, enactment dates, and enactment mechanisms (via executive order or through a legislative bill) is presented in Table 1.

The outcome of interest was county-level, weekly, first-dose COVID-19 vaccinations among persons <65 years old, as those 65 and older were less likely to report vaccine resistance [2,3]. We obtained data from the Centers for Disease Control and Prevention’s (CDC) publicly available COVID-19 data repository and supplemented missing data with state health department data [29]. We calculated weekly, first-dose COVID-19 vaccinations by summing daily first-dose COVID-19 vaccinations over a Monday–Sunday period, with week of enactment considered “Week 0” and included in the pre-period. However, even aggregated weekly COVID-19 vaccinations seemed to suffer from lags in data collection and reporting. To smooth out weekly values, our primary analysis aggregated weekly, first-dose vaccination estimates following POV mandate ban enactment into bimonthly (every two weeks) time periods. We also conducted sensitivity analysis on weekly and monthly effects post-POV mandate ban (Appendix A).

Data for other time-varying covariates, including other state vaccine policies and COVID-19 deaths, was collected from the CDC’s COVID-19 data repository and the NASHP website [7,30]. County and city vaccine policies were collected from the HHS, and data for sensitivity testing was collected from US Census and CDC Hesitancy Data [31,32,33].

### 2.3. Study Sample

We used a geographic regression discontinuity (GRD) design to determine our study sample, which included treatment and control counties within 150 miles of the POV mandate ban state border. The GRD design was sharp, and the running variable was county centroid distance, in miles, from the state border. GRD design is a quasi-experimental method which exploits “local randomization” that occurs when an arbitrary cut-off point, such as a state border, determines treatment/control samples. The design speculates that counties close to the POV mandate ban state border will be similar in factors related to COVID-19 vaccination, which is supported by studies investigating spatial proximity on vaccine uptake [34]. This selection mechanism has been used in other COVID-19 policy analyses and was also empirically tested in our sample (Appendix A) [35,36].

This sample selection excluded many counties (and the whole state of AK) that do not share a state border with a POV mandate ban state, were too far away from the state border to be considered comparable controls, and/or had no US border counties (i.e., counties along the Canadian border, counties along the Gulf of Mexico). This selection mechanism also resulted in some counties acting as multiple controls. We conducted additional sensitivity testing that included all counties within selected states and that removed multiple county controls (Appendix A).

In addition to the GRD design, we also used a stacked data structure, which removed early vs. late adopter county comparisons and can be problematic in two-way fixed-effects estimation (TWFEE) [37]. We also removed time periods for states that enacted multiple POV mandate bans during the study period, which included FL, MT, AZ, ND, SC, and GA. Both the stacked structure and removal of secondary POV mandate bans led to an unbalanced panel data set.

Finally, we excluded counties for dates that had no data on first-dose COVID-19 vaccinations for persons <65 years old (8.4% of sample). This missing data occurred throughout the study sample, with 88.9% occurring in Nebraska (NE), SD, TX, New Mexico (NM) and Ohio (OH). The missingness was almost evenly split among treatment and control, with 53% of missing observations occurring in treatment counties and 48% occurring in control counties. Our final sample included 4612 observations (with duplicate controls) and 2466 unique counties. GIF S1 in the Supplementary Files depicts how our sample changed over time.

### 2.4. Study Period

The first POV mandate bans for each state were enacted between 16 March 2021 and 29 September 2021. Based on previous COVID-19 vaccination studies, we used dates up to eight weeks pre-POV mandate ban enactment and 12 weeks post-POV mandate ban enactment [8,9]. For UT counties and controls, we only extended dates up to five weeks pre-POV mandate ban due to early data missingness and inaccessibility to COVID-19 vaccinations in early 2021. We also conducted sensitivity analysis of varying inclusion criteria for weeks before the POV mandate ban enactment date with similar results (Appendix A). With pre- and post-period designations included, our study time frame spans from 8 February 2021 to 25 October 2021.

### 2.5. Sample Stratification

Our sample includes all first POV mandate bans passed in 2021, but we do not believe that POV mandate bans were a monolith. Instead, POV mandate bans enacted through executive order versus the legislative process had some crucial differences that we believe signaled different social norms to the population through the EFL. Among these are potential differences in content and language for a governor passing a relatively quick executive order vs. state congress members that needed a majority to approve the legislation [38]. In addition to analyzing the full sample of POV mandate bans, we also stratified our analysis by executive order and legislative POV mandate bans (termed full sample, executive order sample, and legislative samples in Section 3 and Section 4). Ten states passed POV mandate bans through executive orders and 11 passed POV mandate bans through the legislative process [7].

### 2.6. Statistical Analysis

We estimated the effect of POV mandate ban enactment on the log of weekly, first-dose COVID-19 vaccinations using a stacked TWFEE framework and a GRD design to select treatment/control groups. We selected a Gaussian distribution for our TWFEE model, but checked that result significance was similar under Poisson and negative binomial distributions. The TWFEE is a type of difference-in-differences (DiD) analysis and allowed us to study county trends before and after POV mandate ban enactment against controls that did not experience POV mandate ban enactment during the same time. The combination of GRD along with the TWFEE allowed us to select comparable treatment and control counties and eliminate state-border confounding (i.e., different healthcare systems) and secular trends. Below is the equation for our model:

(1) $$\begin{array}{c} Y_{ct}=\alpha_{c}+\alpha_{t}+\beta_{1}D_{c}+\beta_{2}T_{post=1,tp}+\sum_{p=1}^{6}\delta_{p}\left(T_{post=1,tp}*D_{c}\right)+X_{ct}\beta^{\prime }+\epsilon_{ct} \end{array}$$

where *c* is county and *t* is time in weeks. $Y_{ct}$ is the log of weekly, first-dose COVID-19 vaccinations, $\alpha_{t}$ are time-fixed effects (using dummy variables for date), $\alpha_{c}$ are county-fixed effects, and $X_{ct}\beta^{\prime }$ is the vector of time-variant covariates. The time-varying covariates included county centroid distance to the POV mandate ban state border; vaccine-specific state, county, or city legislation; one-week COVID-19 death rate lag; and a one-week lag for the percent of weekly averaged, cumulative first-dose vaccinations among persons <65 years old. Variable $T_{post=1,tp}$ represents the weeks following POV mandate ban enactment at time *t*, aggregated bimonthly (*p*), and $D_{c}$ is a dummy variable for treatment/control counties. The coefficients of interest were the set of interaction terms, $\delta_{p}$, which we interpreted as the additional percent change in weekly, first-dose COVID-19 vaccinations for aggregated bimonthly time periods, *p*, among treatment counties, before and after POV mandate ban enactment when compared control counties. In our Section 3 we present aggregated, bimonthly coefficients ($\delta_{1-6}$), standard errors (s.e.) for those coefficients, and *p*-values. All analyses were performed in Stata/SE 19.5 and standard errors were clustered at the state level.

## 3. Results

**Figure 2:**
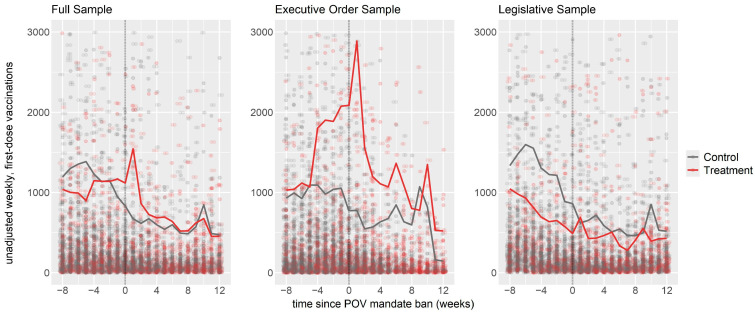
Unadjusted weekly, first-dose COVID-19 vaccinations for persons under 65 years old before and after POV mandate ban enactment for full, executive order, and legislative samples, respectively.

To explore changes in COVID-19 vaccinations during POV mandate ban enactments, we first looked at unadjusted weekly, first-dose COVID-19 vaccinations (Figure 2). We saw an increase in COVID-19 vaccinations among treatment counties following POV mandate ban enactment for all three samples and no increase in control counties. The full sample spike in COVID-19 vaccinations was likely due to the large increase seen in the executive order sample, as the legislative sample had only a small increase. Of note, there was an increase in unadjusted COVID-19 vaccinations around four weeks before the POV mandate ban enactment among the executive order sample. This was due to the introduction of Texas counties, which did not have data on first-dose COVID-19 vaccinations among those <65 years old before 2 March 2021.

For our adjusted model outcomes, we found no significant effect on the percent change in weekly, first-dose COVID-19 vaccinations for the full POV mandate ban sample (Table 2). However, when we stratified the sample, we found a significant, positive effect in the first several time periods post-POV mandate ban among the executive order sample. We saw an additional 38.2% increase in Weeks 1–2, 40.6% in Weeks 3–4, 41.3% in Weeks 5–6, and 43.9% in Weeks 7–8 post enactment. Among the legislative sample, no time periods had significant percent changes in weekly, first-dose COVID-19 vaccinations when compared to control counties. Parallel trend testing was satisfied for both the executive order and legislative samples after adjusting for time-varying confounding (Table A1; Appendix A).

**Figure 3:**
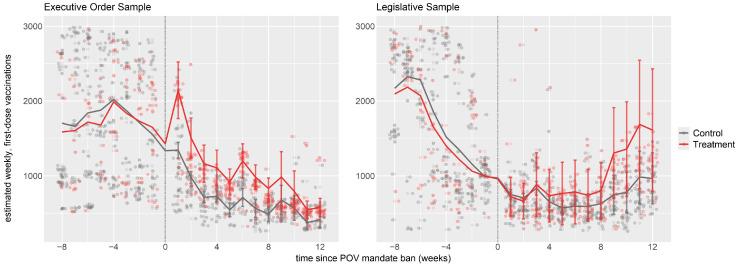
Predicted TWFEE values for weekly, first-dose COVID-19 vaccinations before and after POV mandate ban enactment for executive order and legislative samples, respectively.

Figure 3 shows estimated weekly, first-dose COVID-19 vaccinations before and after POV mandate ban enactment for both executive order and legislative samples. We saw that within the executive order sample, treatment counties had a significant spike in first-dose COVID-19 vaccinations that remained significantly above control counties even as both trended downward. The range of additional percent increases among treatment counties (38.2–43.9%) corresponded to an estimated additional increase of 415–699 weekly, first-dose COVID-19 vaccinations.

We also conducted additional model specifications that included varying inclusion distances to the POV mandate ban state border and removed spillover counties (Appendix A). These sensitivity tests confirmed the results of our primary analysis. Further, we tested the main model specification with different assumptions including non-normal distributions, Sun and Abrahams TWFEE corrections, TWFEE with Poisson and negative binomial distributions, and unspecified nonparametric model fixed effects [39]. While estimates and standard errors differed, all models showed an additional percent increase in weekly, first-dose COVID-19 vaccinations for the first several bimonthly time periods post-POV mandate ban when compared to control counties for the executive order sample.

## 4. Discussion

Among the executive order sample, we found an additional increase in the percent change in weekly, first-dose COVID-19 vaccinations post-POV mandate ban when compared with controls and no effect among the legislative sample. We believe that the EFL combined with the PRT was the primary mechanisms for behavior change, which signaled a protection of individual liberties for residents experiencing reactance behavior to vaccine mandates. If our hypothesis was accurate, the effect would occur amongst areas with higher populations of documented COVID-19 resistance and strongly held beliefs around individual liberties, as these groups would likely have the highest reactance to news and media around vaccine mandates [2]. To check this, we further sectioned our samples (at the sample mean) for the following variables associated with COVID-19 vaccine resistance; percent of the county voting for Trump in 2020 (Republican), percent of the county that reported strong COVID-19 hesitancy in Spring 2021 (estimated by the CDC using the Census Household Survey), percent of the county with a college education, and percent of the county that is uninsured [32]. We found that the increased effect among the executive order sample was concentrated among communities with more Trump voters, higher COVID-19 vaccine hesitancy, fewer college degrees, and more uninsured people (Table 3) [31,32].

However, this does not explain the more robust signal among the executive order sample. When reviewing the bills, we found two distinctions: (1) on average, executive order POV mandate bans included more public and private sector organizations and were more focused on COVID-19 vaccinations, and (2) the language among executive orders was more explicit about individual liberties and government overreach. Our descriptive review found that 60% of executive orders also included private businesses with and without public funding as opposed to only 18% of legislative POV mandate bans. Further, 45% of legislative POV mandate bans were components of other legislative periodic bills, such as omnibus spending bills, and were not their own legislative bill [7]. This would result in a diluted signal as compared to executive orders which were solely focused on the POV mandate ban.

Regarding the bill’s language, Table 4 provides some quotes from executive order and legislative POV mandate bans. Executive orders tended to explicitly address the importance of individual liberties, that vaccine mandates/passports threaten those liberties, and the need to protect those liberties through POV mandate bans. Further, the language used in executive orders mirrors conservative political rhetoric at the time and this connection could result in a stronger signal to constituents experiencing reactionary behavior caused by the same political rhetoric [40]. Conversely, legislative POV mandate bans mostly just detailed the procedures and functions of the POV mandate ban. In cases where legislative POV mandate bans did use language around individual liberties to cite reasons for enactment, the intensity was muted compared to executive orders.

### Limitations

There were limitations with our analysis. With any DiD specification, there is the potential for other events to affect the relationship between the treatment and outcome. We were able to control for some city- and state-level vaccine policies; however, it is possible that these data were not exhaustive [33]. Further, roughly 8% of the full sample was missing, which reduces the generalizability of the effect we found among the executive order sample. We also further reduced generalizability by selecting a sample and study period that would maximize the internal validity of our analysis. However, even under a restricted sample, our executive order analysis was representative of 39% of all US counties and 44% of contiguous American states. While not generalizable to the entire US population, we believe it was sufficiently representative of Americans who were resistant of COVID-19 vaccinations due to reactance to vaccine mandates. Finally, because we could not measure person-level perceptions or when constituents were made aware of the POV mandate ban enactment, we are unable to know for certain which persons were motivated by reversal of reactance. However, our study design leveraged multiple quasi-experimental approaches, performed several sensitivity analyses on various model specifications and sub-group populations, and reviewed the content and language within the POV mandate bans. The diversity of testing and sensitivity checks performed gives us more confidence in our conclusions.

## 5. Conclusions

POV mandate bans, specifically executive orders, increased weekly, first-dose COVID-19 vaccinations among those <65 years old following POV mandate ban enactment across counties with a high propensity for reactance. The mechanism for these results, under the EFL and PRT frameworks, is more straightforward than without these conceptual models for context. Under an increasing polarizing time for politics, conservative politics emphasized the importance of individuals liberties and the detriment of vaccine mandates, while democratic politics engaged in messaging and policy that PRT studies directly warn against [17,21,41]. POV mandate bans removed the perceived threat perpetuated by conservative media, signaled existing community values around individual liberties, and mitigated reactance behavior.

The downstream effects of vaccine reactance go beyond POV mandate bans. Research has shown that subsequent voluntary uptake of COVID-19 boosters was less among states with vaccine mandates than states with POV mandate bans, making them more susceptible to future COVID-19 strains [42]. We are now experiencing the large-scale implications of vaccine reactance with elected officials at HHS removing experts on immunizations and changing mandatory vaccine schedules [43].

While it may be easy to conclude that POV mandate bans were innocuous or even beneficial for vaccination rates, POV mandate bans set a dangerous precedent for both public health and policy. POV mandate bans were masked preemption policies, with some targeting the scope of both local and federal governments to enforce vaccinations [38]. This scope extends to areas, such as hospitals and schools, where vaccination requirements have historically been supported and are considered standard practice [7,44].

As we consider the implications of this research for future emergencies, it is important to understand that POV mandate ban effects were the result of a unique climate. The COVID-19 pandemic was at the intersection of unforeseen political polarization in America and the advent of social media promoting widespread mis- and dis-information, all combined during a once-in-a-century pandemic [45,46,47]. Given the multifaceted nature of this problem, we suggest that public health officials and public policy experts evaluate mandatory policies and messaging within their specific contexts. We believe that vaccine mandates are a potentially necessary and powerful public health measure, but only meaningful in the US when reactance behaviors are avoided by ensuring constituents’ liberty concerns are assuaged and values upheld.

## Figures and Tables

**Table 1:** Details on policy mechanism (passed via executive order or through house and senate bills), date of enactment, and state proof-of-vaccine (POV) mandate bans for the first POV mandate bans during the 2021 calendar year [7].

State	Enactment Date	Policy Mechanism
Utah (UT)	16 March 2021	HB ^a^ 308
Florida (FL)	2 April 2021	EO ^b^ 2021-81
Texas (TX)	5 April 2021	EO GA-35
Idaho (ID)	7 April 2021	EO 2021-04
Montana (MT)	13 April 2021	EO 7-2021
Arizona (AZ)	19 April 2021	EO 2021-09
South Dakota (SD)	20 April 2021	EO 2021-08
Alaska (AK) ^c^	26 April 2021	AO ^d^ 321
Indiana (IN)	29 April 2021	HB 1405
Arkansas (AR)	30 April 2021	SB ^e^ 615
North Dakota (ND)	7 May 2021	HB 1465
Wyoming (WY)	7 May 2021	EO
South Carolina (SC)	11 May 2021	EO 2021-23
Alabama (AL)	17 May 2021	SB 267
Iowa (IA)	20 May 2021	HF ^f^ 889
Georgia(GA)	25 May 2021	EO 5.25.21.01
Kansas (KS)	26 May 2021	SB 159
Tennessee (TN)	26 May 2021	SB 858
Oklahoma (OK)	1 June 2021	EO 2021-16
Missouri (MO)	15 June 2021	HB 271
New Hampshire (NH)	23 July 2021	HB 220
Michigan (MI)	29 September 2021	SB 82

^a^ HB (House Bill). ^b^ EO (executive order). ^c^ AK was removed since it is not part of the contiguous 48 states and would likely skew results if we were to include “comparable control” counties based on distance. ^d^ AO (Administrative Order). ^e^ SB (Senate Bill). ^f^ HF (House File).

**Table 2:** Two-way fixed-effects estimation (TWFEE) coefficients and standard errors (se) for percent (%) change in weekly, first-dose COVID-19 vaccinations post-proof-of-vaccine (POV) mandate ban enactment, aggregated bimonthly, across full, executive order, and legislative samples.

	Full Sample		Executive Order Sample		Legislative Sample	
	Coefficient (se)	*p*-Value	Coefficient (se)	*p*-Value	Coefficient (se)	*p*-Value
Variables						
Treatment Counties × post-POV Mandate Ban periods ($\delta_{p}$)						
Pre-period (ref)	--	--	--	--		
Weeks 1–2	10.2% (12.5%)	0.42	38.2% (10.5%)	0.003 **	−5.7% (14.7%)	0.71
Weeks 3–4	16.7% (12.4%)	0.19	40.6% (12.2%)	0.006 **	3.3% (18.9%)	0.85
Weeks 5–6	16.8% (16.8%)	0.32	41.3% (16.0%)	0.027 *	7.8% (24.3%)	0.73
Weeks 7–8	14.5% (19.7%)	0.46	43.9% (17.3%)	0.030 *	3.5% (27.2%)	0.89
Weeks 9–10	25.1% (23.0%)	0.29	30.2% (18.3%)	0.13	21.5% (29.7%)	0.46
Weeks 11–12	21.2% (25.2%)	0.40	31.9% (15.2%)	0.06	20.4% (32.5%)	0.51

* *p*-value significant at the 0.05 level, ** 0.01 level, and *** <0.001 level.

**Table 3:** Two-way fixed-effects estimation (TWFEE) coefficients and standard errors (se) for percent (%) change in weekly, first-dose COVID-19 vaccinations post-proof-of-vaccine (POV) mandate ban enactment, aggregated bimonthly, for the executive order sample stratified at the sample mean across % of the county voting for Trump in 2020, % of the county with a college degree, % of the county that is uninsured, and % of the county estimated to be strongly COVID-19 vaccine-hesitant.

	% of County Voting for Trump 2020			
	≤69.6%		>69.6%	
	Coefficient (se)	*p*-Value	Coefficient (se)	*p*-Value
Treatment Counties × post-POV Mandate Ban periods ($\delta_{p}$)				
Pre-period (ref)	--	--	--	--
Weeks 1–2	35.1% (16.3%)	0.06	44.8% (9.9%)	0.001 **
Weeks 3–4	43.0% (14.4%)	0.013 *	45.7% (12.4%)	0.003 **
Weeks 5–6	41.8% (17.1%)	0.035 *	49.1% (14.9%)	0.008 **
Weeks 7–8	41.4% (21.0%)	0.08	51.9% (14.7%)	0.005 **
Weeks 9–10	55.5% (27.4%)	0.08	20.6% (10.7%)	0.08
Weeks 11–12	38.5% (22.2%)	0.12	29.8% (12.0%)	0.029 *
	**% of County with College Degree**			
	≤18.4%		>18.4%	
	Coefficient (se)	*p*-value	Coefficient (se)	*p*-value
Treatment Counties × post-POV Mandate Ban ($\delta_{p}$)				
Pre-period (ref)	--	--	--	--
Weeks 1–2	29.1% (9.5%)	0.009 **	51.7% (13.6%)	0.003 **
Weeks 3–4	45.8% (11.4%)	0.002 **	29.3% (15.2%)	0.08
Weeks 5–6	55.7% (15.3%)	0.004 **	22.2% (18.5%)	0.25
Weeks 7–8	53.5% (16.0%)	0.007 **	27.1% (21.0%)	0.22
Weeks 9–10	38.1% (18.4%)	0.07	18.7% (22.9%)	0.41
Weeks 11–12	43.9% (17.8%)	0.034 *	15.9% (15.0%)	0.30
	**% of County Uninsured**			
	≤13.7%		>13.7%	
	Coefficient (se)	*p*-value	Coefficient (se)	*p*-value
Treatment Counties × post-POV Mandate Ban periods ($\delta_{p}$)				
Pre-period (ref)	--	--	--	--
Weeks 1–2	15.7% (12.5%)	0.23	54.2% (12.3%)	0.001 **
Weeks 3–4	24.5% (17.1%)	0.18	58.6% (14.0%)	0.002 **
Weeks 5–6	20.0% (14.4%)	0.19	65.2% (15.8%)	0.002 **
Weeks 7–8	16.0% (23.4%)	0.49	72.9% (15.9%)	0.001 **
Weeks 9–10	18.0% (25.2%)	0.47	26.8% (12.7%)	0.06
Weeks 11–12	3.3% (17.6%)	0.84	64.0% (18.1%)	0.007 **
	**% of County Strongly Hesitant of COVID-19 Vaccines**			
	≤9.36%		>9.36%	
	Coefficient (se)	*p*-value	Coefficient (se)	*p*-value
Treatment Counties × post-POV Mandate Ban periods ($\delta_{p}$)				
Pre-period (ref)	--	--	--	--
Weeks 1–2	10.4% (16.1%)	0.51	41.5% (13.9%)	0.016 *
Weeks 3–4	7.4% (42.0%)	0.84	57.1% (16.4%)	0.008 **
Weeks 5–6	35.8% (49.2%)	0.45	128.2% (10.8%)	<0.001 ***
Weeks 7–8	23.0% (29.0%)	0.43	89.7% (19.6%)	0.002 **
Weeks 9–10	40.0% (27.5%)	0.18	63.2% (16.9%)	0.006 **
Weeks 11–12	37.3% (27.5%)	0.21	40.4% (16.8%)	0.042 *

* *p*-value significant at the 0.05 level, ** 0.01 level, and *** <0.001 level.

**Table 4:** Examples of language used in executive order and legislative proof-of-vaccine (POV) mandate bans.

State	POV Mandate Ban Quotes	Legislation Type
AR	“(a) As used in this section, ‘vaccine passport’ means documentation that an individual has been vaccinated against coronavirus 2019 (COVID-19)”. “(b) The state, a state agency or entity, a political subdivision of the state, or a state or local official shall not require an individual to use a vaccine passport in this state for any purpose.”	SB ^a^ 615
FL	“WHEREAS, requiring so-called COVID-19 vaccine passports for taking part in everyday life—such as attending a sporting event, patronizing a restaurant, or going to a movie theater—would create two classes of citizens based on vaccination…” “WHEREAS, so-called COVID-19 vaccine passports reduce individual freedom and will harm patient privacy…” “WHEREAS, it is necessary to protect the fundamental rights and privacies of Floridians and the free flow of commerce within the state.”	EO ^b^ 2021-81
GA	“WHEREAS: I have determined that the following actions are necessary and appropriate to protect the individual liberty of Georgia’s residents…”	EO 5.25.21.01
KS	“…no state agency named… shall expend any moneys appropriated… to (1) Issue a COVID-19 vaccination passport to any individual without the individual’s consent; (2) require an individual to use a COVID-19 vaccination passport within this state for any purpose…”	SB 159
ND	“A private business located in this state may not require a patron or customer to provide any documentation certifying vaccination… to gain access to, entry upon, or services from the business.” “This section may not be construed to interfere with an individual’s rights to access that individual’s own personal health information…”.	HB ^c^ 1465
SD	“Whereas, The vaccines have led to discussions of ‘vaccine passports’…used to ‘allow’ certain exercises of freedom that Americans already possess …”. “Whereas, Any rationale for imposing public health restrictions that limit freedoms should be tailored to mitigate a verifiable, scientific risk…”. “Whereas, It is improper to adopt an official government policy—a mandate—requiring widespread use of vaccine passports when such a mandate is overreaching, morally objectionable…”.	EO 2021-08

^a^ SB (Senate Bill). ^b^ EO (executive order). ^c^ HB (House Bill).

**Figure A1:**
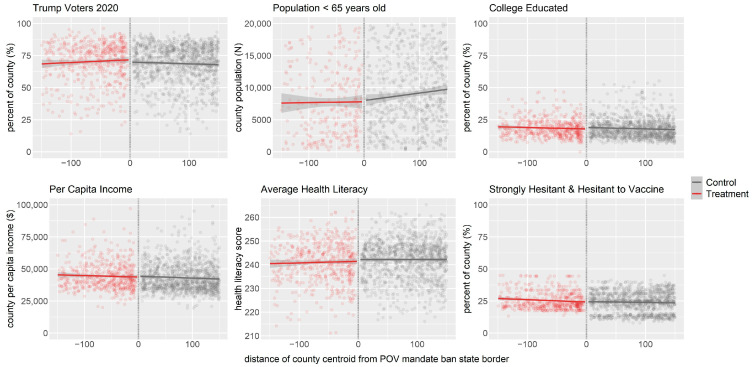
Geographic regression discontinuity (GRD) design graphs showing continuity across the threshold of the running variable, county centroid distance, in miles, from the proof-of-vaccine (POV) mandate ban state border, among confounding factors for the executive order sample.

## Data Availability

Data sourced for this research was publicly available from secondary data sources. A link to a compiled data repository for this analysis can be provided upon request.

## Supplementary Materials

The following supporting information can be downloaded at https://www.mdpi.com/article/10.3390/vaccines14060486/s1. GIF S1. US map depicting changes in the stacked, panel sample of POV mandate ban treatment and control counties over time from 16 March 2021 to 25 October 2021.

## Abbreviations

The following abbreviations are used in this manuscript:

POV	Proof of Vaccine
SEM	Social Ecological Model
EFL	Expressive Function of Law
PRT	Psychological Reactance Theory
GRD	Geographic-Regression Discontinuity
TWFEE	Two-Way Fixed-Effects Estimation
DiD	Difference-in-Differences
HHS	The US Department of Health and Human Services
NASHP	National Academy for State Health Policy
EO	Executive Order
SB	Senate Bill
HB	House Bill

## Appendix A

### Appendix A.1. Parallel Trends

Table A1 shows the insignificant change in weekly, first-dose COVID-19 vaccinations for the interaction between time pre-POV mandate ban and treatment status (rows under “Treatment Status × Time till POV Mandate Ban [in weeks]”). Parallel trends were satisfied for the full, executive order, and legislative samples. We also tested parallel trends with a quadratic function for time and still found that treatment and control counties had similar trends in the pre-POV mandate ban period.

**Table A1:** Parallel trend testing across full, executive order, and legislative models.

	Full Sample		Executive Order Sample		Legislative Sample	
	Coefficient (se)	*p*-Value	Coefficient (se)	*p*-Value	Coefficient (se)	*p*-Value
Variables						
Treatment Status						
Control (ref)	--	--	--	--	--	--
Treatment	−2.1% (8.8%)	0.82	−2.46% (12.4%)	0.085	−10.3% (10.9%)	0.35
Time till POV Mandate Ban (in weeks)	−0.1% (0.1%)	0.92	0.8% (1.7%)	0.63	0.7% (0.4%)	0.13
Treatment Status × Time till POV Mandate Ban (in weeks)						
Control × Time = 0 (ref)	--	--	--	--	--	--
Treatment × Time	−0.5% (1.3%)	0.73	−1.37% (2.7%)	0.62	−2.0% (1.4%)	0.89

* *p*-value significant at the 0.05 level, ** 0.01 level, and *** <0.001 level.

### Appendix A.2. Sample Selection

Our main analysis study sample was selected through a geographic regression discontinuity (GRD) design, which identifies treatment and control counties based on distance from the POV mandate ban state’s border. Based on previous research, we selected a 150-mile buffer from the POV mandate ban state border [35]. GIF S1 in the Supplementary Files shows how the treatment and control sample changes over the study period. We also wanted to test the validity of the GRD design in appropriately selecting treatment and control counties. Figure A1 shows linear estimates for the running variable, county centroid distance (in miles) to POV mandate ban state border, across several potential confounding variables within the executive order sample. The graphs were smooth across the threshold for all tested variables, which is an indication of a valid GRD design. These GRD checks gave us more confidence that our treatment and control counties were similar across factors that have been shown to be related to COVID-19 vaccine acceptance [2].

The main analysis sample also included duplicate control counties for many states who passed POV mandate bans. Depending on their prevalence, they could have disproportionate weight on the results. We conducted sensitivity testing where we remove duplicate counties and found that treatment counties in executive order POV mandate ban states had significantly higher percent increases in weekly, first-dose COVID-19 vaccinations up through Weeks 9–10 post-POV mandate ban when compared to control counties.

Further, we also analyzed the effect of POV mandate bans using a “full-county” sample (not to be confused with the full sample in the main analysis that combined executive order and legislative samples). The full-county sample included all counties within treatment and control states that had complete weekly, first-dose COVID-19 vaccination data. We still selected only neighboring states because of the staggered policy adoption with POV mandate bans and removed neighboring states that had already enacted a POV mandate ban to control for early vs. late adopter comparisons [37]. In the full-county sample, only Weeks 1–2 post-POV mandate ban enactment among executive order counties had significant additional increases in weekly, first-dose COVID-19 vaccinations when compared to control counties ($\delta_{1}$ = 32.6%; *p*-value = 0.022). Table A2 shows the TWFEE results for executive order and legislative POV mandate ban full-county samples.

**Table A2:** Two-way fixed-effects estimation (TWFEE) coefficients and standard errors (se) for percent (%) change in weekly, first-dose COVID-19 vaccinations, aggregated bimonthly, across executive order and legislative proof-of-vaccine (POV) mandate bans for full-county samples.

	Executive Order Sample		Legislative Sample	
	Coefficient (se)	*p*-Value	Coefficient (se)	*p*-Value
Variables				
Treatment Counties × post-POV Mandate Ban periods ($\delta_{p}$)				
Pre-period (ref)	--	--	--	--
Weeks 1–2	32.6% (12.3%)	0.022 *	1.2% (19.6%)	0.95
Weeks 3–4	22.8% (13.1%)	0.11	11.1% (19.9%)	0.57
Weeks 5–6	22.5% (19.2%)	0.26	22.5% (27.2%)	0.41
Weeks 7–8	28.1% (19.8%)	0.18	13.7% (27.6%)	0.60
Weeks 9–10	4.3% (19.7%)	0.82	22.2% (31.6%)	0.47
Weeks 11–12	18.6% (16.6%)	0.28	22.4% (34.4%)	0.50

* *p*-value significant at the 0.05 level, ** 0.01 level, and *** <0.001 level.

However, we believe the estimates for later bimonthly time periods are no longer significant among executive order counties, because the full-county sample included counties that were not comparable to those in POV mandate ban states. This was shown when looking at characteristic differences between treatment and control counties for the main model GRD sample and the full-county sample (Table A3). Further, our GRD design selection mechanism relies on evidence showing spatial proximity as predictors of vaccine uptake and removes investigator bias and potential unknown confounding that may bias other methods for selecting comparable treatment and control groups (i.e., propensity score matching) [34].

**Table A3:** Averages and *t*-test *p*-values for time-constant factors related to COVID-19 vaccinations for geographic regression discontinuity (GRD) and full-county samples.

	GRD Sample		*p*-Value	Full-County Sample		*p*-Value
	Treatment	Control		Treatment	Control	
Trump votes in 2020 (% of county)	69.6%	64.5%	<0.001 ***	69.4%	60.9%	<0.001 ***
Population Size (<65-year-olds; N)	67,078	73,091	0.59	68,670	97,750	0.012 *
Health Literacy Scores	243.6	244.0	0.22	242.5	244.2	<0.001 ***
Proportion ≥ 65 years old (%)	18.6%	18.7%	0.67	18.8%	18.8%	0.94
College Degree (%)	18.3%	19.2%	0.006 **	18.1%	20.2%	<0.001 ***
Per Capita Income 2019 ($)	$44,692	$44,818	0.79	$44,367	$46,250	<0.001 ***

* *p*-value significant at the 0.05 level, ** 0.01 level, and *** <0.001 level.

### Appendix A.3. Study Period

In addition to testing various study samples, we also tested various study periods with a specific emphasis on various pre-period inclusions. We limited our main analysis study period to eight weeks pre-POV mandate ban enactment, with the exception of UT whose pre-period only extended to five week. This was to accommodate early POV mandate ban adopters with pre-periods extending into January 2021, which had limited data availability and COVID-19 vaccination access for persons <65 years old and would present a downward bias for these POV mandate ban comparisons. Further, for those counties with data, the population <65 years old that had access to COVID-19 vaccines in January 2021 were essential workers, immunocompromised, and not representative of the general population [48]. However, when testing this in the executive order sample, we still found that the significant positive effects remained for the first two time periods post-POV mandate ban when including up to 12 weeks of pre-POV mandate ban enactment (Weeks 1–2, $\delta_{1}$ = 30.4%, standard error (se) = 10.3%, *p*-value = 0.012; Weeks 3–4, $\delta_{2}$ = 33.9%, se = 12.4%, *p*-value = 0.018). This was also true when restricting the study pre-period up to four weeks pre-POV mandate ban enactment.

### Appendix A.4. Sensitivity Testing

We performed many sensitivity analyses on our main model specification, including the tests shown above to determine our sample and study period. In addition, we also looked at various aggregated post-time periods, including weekly and monthly values (Table A4). The main model specifications looked at bimonthly time periods post-POV mandate ban to smooth out delays in reporting and inaccuracies that were present even when vaccination data was aggregated to weekly values. Table A3 shows the interaction term $\delta_{p}$ with weekly time periods (n = 12) for treatment counties. The weekly time periods for the executive order sample showed alternating significance for the percent change in weekly, first-dose COVID-19 vaccinations and no change in significance for the legislative sample. Part of our rationale to aggregate weekly values into bimonthly time periods was due to the inconsistent nature of COVID-19 vaccination reporting in the first half of 2021 and the natural staggering that may have occurred as constituents made appoints for first-dose COVID-19 vaccination [29]. Further, when aggregated into monthly time periods, we saw significant estimates similar to those when using bimonthly time periods (Table A5).

**Table A4:** Two-way fixed effects estimation (TWFEE) coefficients and standard errors (se) for percent (%) change in weekly, first-dose COVID-19 vaccinations across executive order and legislative proof-of-vaccine (POV) mandate ban samples using weekly time periods.

	Executive Order Sample		Legislative Sample	
	Coefficient (se)	*p*-Value	Coefficient (se)	*p*-Value
Variables				
Treatment Counties × post-POV Mandate Ban periods ($\delta_{p}$)				
Pre-period (ref)	--	--	--	--
Week 1	61.2% (13.5%)	0.001 **	−2.0% (14.8%)	0.90
Week 2	18.4% (18.3%)	0.32	−9.2% (17.1%)	0.61
Week 3	62.8% (15.4%)	0.002 **	−5.6% (14.4%)	0.71
Week 4	17.4% (16.8%)	0.31	13.2% (22.0%)	0.54
Week 5	47.3% (17.8%)	0.025 *	28.3% (27.4%)	0.31
Week 6	34.2% (14.2%)	0.035 *	−9.3% (21.4%)	0.69
Week 7	37.6% (16.6%)	0.047 *	−8.3% (22.3%)	0.73
Week 8	51.7% (19.6%)	0.027 *	17.1% (27.9%)	0.52
Week 9	13.4% (22.5%)	0.54	41.7% (29.4%)	0.18
Week 10	48.2% (19.1%)	0.032 *	5.0% (36.1%)	0.87
Week 11	20.9% (15.1%)	0.19	21.6% (33.4%)	0.50
Week 12	43.7% (16.7%)	0.026 *	19.8% (32.4%)	0.52

* *p*-value significant at the 0.05 level, ** 0.01 level, and *** <0.001 level.

**Table A5:** Two-way fixed effects estimation (TWFEE) coefficients and standard errors (se) for percent (%) change in weekly, first-dose COVID-19 vaccinations across executive order and legislative POV mandate ban samples using monthly time periods.

	Executive Order Sample		Legislative Sample	
	Coefficient (se)	*p*-Value	Coefficient (se)	*p*-Value
Variables				
Treatment Counties × post-POV Mandate Ban periods ($\delta_{p}$)				
Pre-period (ref)	--	--	--	--
Weeks 1–4	39.4% (10.8%)	0.003 **	−1.3% (13.3%)	0.93
Weeks 5–8	43.8% (16.1%)	0.021 *	5.7% (23.8%)	0.80
Weeks 9–12	28.7% (14.9%)	0.08	20.8% (30.1%)	0.48

* *p*-value significant at the 0.05 level, ** 0.01 level, and *** <0.001 level.

We also conducted sensitivity checks to confirm that our model was robust to varying designated distances when selecting treatment and control counties and robust to potential spillover effects (Table A6 and Table A7). We tested TWFEE for county samples within 125, 100, and 75 miles of the POV mandate ban state border for both legislative and executive order samples. We saw largely the same results as the main model, with the exception that Weeks 5–6 post-POV mandate ban was no longer significantly different for the 100-mile buffer in the executive order sample. We also tested the potential for spillover effects. These sensitivity tests removed counties with county centroid distances within 5, 10, and 25 miles from the POV mandate ban state border. We saw no changes in the estimate’s significance for either executive order or legislative samples, suggesting that our results were robust to potential spillover bias.

**Table A6:** Two-way fixed effects estimation (TWFEE) coefficients and standard errors (se) for percent (%) change in weekly, first-dose COVID-19 vaccination, aggregated bimonthly, across executive order and legislative proof-of-vaccine (POV) mandate ban samples for varying geographic regression discontinuity (GRD) distance buffers.

	125 Miles		100 Miles		75 Miles	
	Coefficient (se)	*p*-Value	Coefficient (se)	*p*-Value	Coefficient (se)	*p*-Value
Executive Order POV Mandate Ban States						
$\mathrm{Interaction}\;\mathrm{Term}\;(\delta_{p}$)						
Weeks 1–2	38.9% (10.4%)	0.002 **	36.9% (10.4%)	0.004 **	39.0% (10.3%)	0.002 **
Weeks 3–4	43.8% (12.1%)	0.003 **	43.4% (13.2%)	0.007 **	50.3% (13.1%)	0.003 **
Weeks 5–6	41.6% (17.0%)	0.035 *	38.5% (18.6%)	0.07	41.4% (17.8%)	0.044 *
Weeks 7–8	48.2% (18.1%)	0.025 *	50.6% (19.2%)	0.027 *	57.0% (18.1%)	0.012 *
Weeks 9–10	40.7% (18.1%)	0.05	34.9% (19.7%)	0.11	44.7% (19.7%)	0.05
Weeks 11–12	47.6% (19.3%)	0.036 *	46.7% (21.4%)	0.06	44.6% (19.6%)	0.049 *
Legislative POV Mandate Ban States						
$\mathrm{Interaction}\;\mathrm{Term}\;(\delta_{p}$)						
Weeks 1–2	−8.6% (13.8%)	0.55	−11.5% (13.3%)	0.40	−11.7% (13.0%)	0.38
Weeks 3–4	0.8% (18.1%)	0.96	−1.3% (15.1%)	0.94	−2.7% (14.4%)	0.86
Weeks 5–6	5.0% (22.9%)	0.82	3.1% (21.9%)	0.88	1.3% (21.3%)	0.95
Weeks 7–8	0.3% (26.5%)	0.99	−1.9% (20.6%)	0.93	−6.7% (20.9%)	0.77
Weeks 9–10	19.8% (28.7%)	0.48	19.4% (28.1%)	0.48	15.0% (28.5%)	0.58
Weeks 11–12	18.8% (32.0%)	0.54	16.8% (31.4%)	0.57	11.6% (31.7%)	0.69

* *p*-value significant at the 0.05 level, ** 0.01 level, and *** <0.001 level.

**Table A7:** Two-way fixed effects estimation (TWFEE) coefficients and standard errors (se) for percent (%) change in weekly, first-dose COVID-19 vaccinations, aggregated bimonthly, across executive order and legislative proof-of-vaccine (POV) mandate ban samples for varying spillover buffer.

	5-Mile Buffer		10-Mile Buffer		25-Mile Buffer	
	Coefficient (se)	*p*-Value	Coefficient (se)	*p*-Value	Coefficient (se)	*p*-Value
Executive Order POV Mandate Ban States						
$\mathrm{Interaction}\;\mathrm{Term}\;(\delta_{p}$)						
Weeks 1–2	38.1% (10.5%)	0.003 **	37.7% (10.2%)	0.003 **	42.3% (10.0%)	0.001 **
Weeks 3–4	40.6% (12.1%)	0.006 **	40.4% (11.7%)	0.005 **	45.9% (12.4%)	0.003 **
Weeks 5–6	41.3% (15.9%)	0.026 *	40.4% (15.8%)	0.028 *	42.2% (15.9%)	0.024 *
Weeks 7–8	43.9% (17.4%)	0.031 *	42.6% (17.3%)	0.034 *	47.1% (16.6%)	0.018 *
Weeks 9–10	30.4% (18.3%)	0.13	28.0% (17.4%)	0.14	30.3% (17.7%)	0.11
Weeks 11–12	32.0% (15.3%)	0.06	32.0% (15.2%)	0.06	33.7% (14.2%)	0.037 *
Legislative POV Mandate Ban States						
$\mathrm{Interaction}\;\mathrm{Term}\;(\delta_{p}$)						
Weeks 1–2	−5.6% (14.9%)	0.72	−4.2% (15.5%)	0.80	0.4% (20.5%)	0.98
Weeks 3–4	3.5% (19.0%)	0.85	6.4% (18.7%)	0.72	10.4% (21.1%)	0.61
Weeks 5–6	7.8% (24.4%)	0.73	10.3% (24.2%)	0.65	14.3% (27.7%)	0.59
Weeks 7–8	3.7% (27.4%)	0.88	6.5% (27.0%)	0.79	8.8% (28.1%)	0.74
Weeks 9–10	21.6% (30.0%)	0.46	23.8% (29.6%)	0.42	25.9% (31.5%)	0.40
Weeks 11–12	20.7% (32.8%)	0.51	24.7% (32.6%)	0.44	27.2% (33.7%)	0.41

* *p*-value significant at the 0.05 level, ** 0.01 level, and *** <0.001 level.
