# Supplementary material for: How Partisan Policies Can Shape Health Behaviors: Executive Order Proof-of-Vaccine Mandate Bans Increased COVID-19 Vaccinations
Source: Vaccines (Basel). 2026 May 29;14(6):486. doi: 10.3390/vaccines14060486 (PMC13307828; doi:10.3390/vaccines14060486)

GIF S1

GIF S1. US map depicting changes in the stacked, panel sample of POV mandate ban treatment and control counties over time from March 16, 2021 – October 25, 2021.

GIF S1 shows our stacked study sample and how it changes over time. The red counties are treatment counties and the grey are control counties. If you download the pdf version and open in Adobe, you can click on the play button at the bottom of the graph to start the GIF.

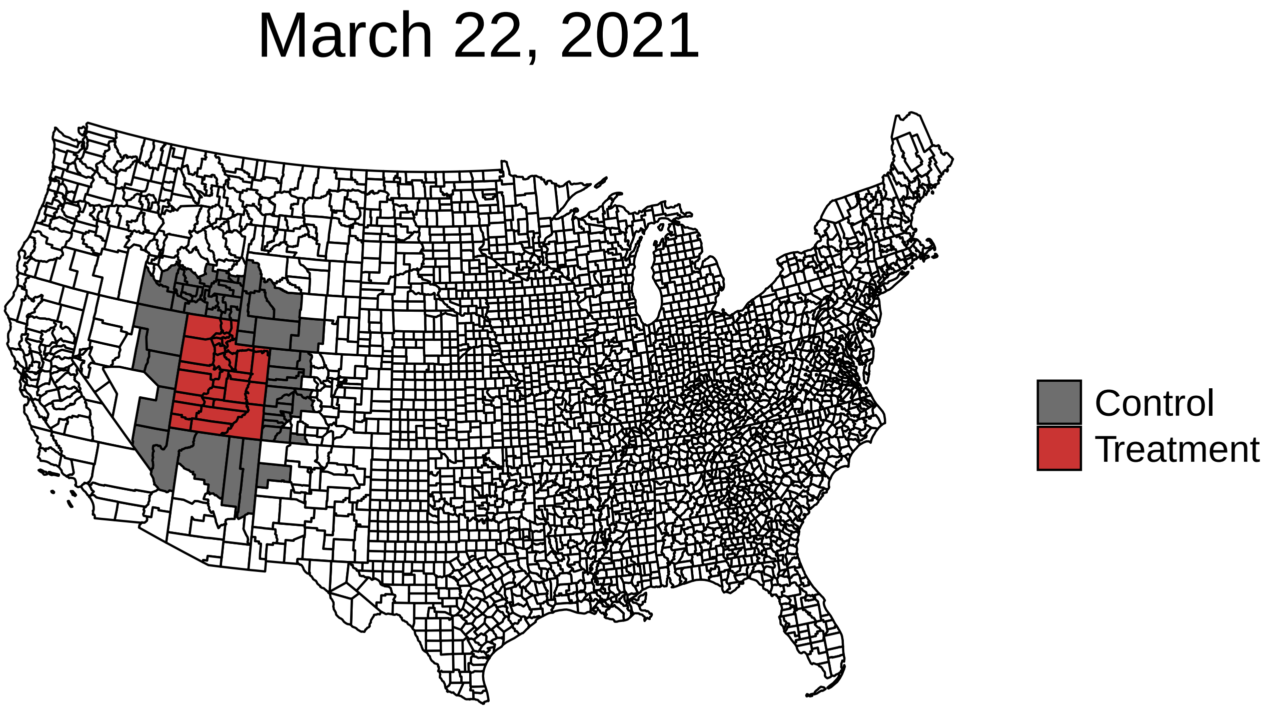

Supplement: Supplementary file 1 [file vaccines-14-00486-s001.zip › vaccines-4287357-supplementary.pdf]
